# Alterations in B Cell and Follicular T-Helper Cell Subsets in Patients with Acute COVID-19 and COVID-19 Convalescents

**DOI:** 10.3390/cimb44010014

**Published:** 2021-12-30

**Authors:** Igor V. Kudryavtsev, Natalia A. Arsentieva, Oleg K. Batsunov, Zoia R. Korobova, Irina V. Khamitova, Dmitrii V. Isakov, Raisa N. Kuznetsova, Artem A. Rubinstein, Oksana V. Stanevich, Aleksandra A. Lebedeva, Evgeny A. Vorobyov, Snejana V. Vorobyova, Alexander N. Kulikov, Maria A. Sharapova, Dmitrii E. Pevtcov, Areg A. Totolian

**Affiliations:** 1 Institute of Experimental Medicine, Akademika Pavlova 12, 197376 Saint Petersburg, Russia; igorek1981@yandex.ru (I.V.K.); issakovd71@gmail.com (D.V.I.); 2 Medical Facutly, First Saint Petersburg State I. Pavlov Medical University, L’va Tolstogo St. 6-8, 197022 Saint Petersburg, Russia; batsunov@gmail.com (O.K.B.); kuznetzova.rais@yandex.ru (R.N.K.); arrubin6@mail.ru (A.A.R.); oksana.stanevich@gmail.com (O.V.S.); aftotrof@gmail.com (A.A.L.); Vorobyeveval@gmail.com (E.A.V.); blaze04@mail.ru (S.V.V.); ankulikov2005@yandex.ru (A.N.K.); kvakva29@gmail.com (M.A.S.); dmitriipevtcov@gmail.com (D.E.P.); totolian@spbraaci.ru (A.A.T.); 3 Laboratory of Immunology, Saint Petersburg Pasteur Institute, Mira 14, 197101 Saint Petersburg, Russia; zoia-korobova@yandex.ru (Z.R.K.); div-o@mail.ru (I.V.K.); 4 Smorodintsev Research Institute of Influenza, Prof. Popov St.15/17, 197376 Saint Petersburg, Russia

**Keywords:** COVID-19, SARS-CoV-2, B cell subsets, T follicular helper cells, Tfh cell subsets, convalescents

## Abstract

Background. Humoral immunity requires interaction between B cell and T follicular helper cells (Tfh) to produce effective immune response, but the data regarding a role of B cells and Tfh in SARS-CoV-2 defense are still sparse. Methods. Blood samples from patients with acute COVID-19 (*n* = 64), convalescents patients who had specific IgG to SARS-CoV-2 N-protein (*n* = 55), and healthy donors with no detectable antibodies to any SARS-CoV-2 proteins (HC, *n* = 44) were analyses by multicolor flow cytometry. Results. Patients with acute COVID-19 showed decreased levels of memory B cells subsets and increased proportion plasma cell precursors compared to HC and COVID-19 convalescent patients, whereas for the latter the elevated numbers of virgin naïve, Bm2′ and “Bm3+Bm4” was found if compared with HC. During acute COVID-19 CXCR3+CCR6− Tfh1-like cells were decreased and the levels of CXCR3−CCR6+ Tfh17-like were increased then in HC and convalescent patients. Finally, COVID-19 convalescent patients had increased levels of Tfh2-, Tfh17- and DP Tfh-like cells while comparing their amount with HC. Conclusions. Our data indicate that COVID-19 can impact the humoral immunity in the long-term.

## 1. Introduction

A pandemic of the novel coronavirus infection called COVID-19 affected millions of people worldwide. The acute period of the COVID-19 may proceed asymptomatically or with diverse clinical manifestations (fever, cough, sore throat, loss of smell and taste, headache, muscle pain, diarrhea, etc.), developing viral pneumonia, hyperinflammation, acute respiratory distress syndrome (ARDS), and multiple organ failure [1]. In the majority of cases, COVID-19 proceeds with few manifestations (about 80% cases), featured with middle course and developing localized inflammation, most often associated with pneumonia and overt respiratory failure that requires less often administration of additional ventilation support [2]. The disease severity may be determined by multiple factors, whereas analysis of the relevant pathogenetic aspects underlying severe COVID-19 revealed that immune cells as well as ACE-2-expressing host cells represent the most crucial cues involved in developing infectious process [3]. In particular, during SARS-CoV-2 infection diverse immune cell types become rapidly activated resulting in upregulated expression of monocyte- [4] and lymphocyte-activation markers [5], emergence of diverse tissue-specific exosomes in peripheral blood [6], as well as increased level of key pro-inflammatory cytokines and acute inflammatory phase proteins [7,8]. A successful interplay between cell and humoral arms of the immune system accounts for efficacy of developing defense reaction against invading pathogens. At the moment, a great body of evidence regarding B cell involvement in response against SARS-CoV-2 has been accumulated, but the data regarding a role for T follicular helper cells (Tfh)—the major T cell subset ensuring regulation of humoral immune response—are still sparse. Tfh cells are necessary for efficient differentiation to plasma cells and memory B cells inside the germinal center of the secondary lymphoid tissues, because they exert several functions (primarily IL-21 production and expression of co-stimulation molecules ICOS and CD40 ligand) necessary for efficient delivery of support during B cell differentiation [9,10].

## 2. Materials and Methods

### 2.1. Patient Characteristics

Our study was performed on the cohort of 119 patients, all of which gave their informed consent to participate in the study, the protocol being approved by the Ethics Committee of the Saint Petersburg Pasteur Institute in accordance with the Declaration of Helsinki. The cohort consisted of patients with present COVID-19 (*n* = 64), convalescents (*n* = 55) without any current symptoms of COVID-19 who had detectable specific IgG antibodies to SARS-CoV-2 nucleocapsid protein, and healthy donors (*n* = 44) without any previous history of COVID-19 and no detectable antibodies to any SARS-CoV-2 protein. All samples were acquired from April to November 2020.

Patients with acute COVID-19 were treated at the First Saint Petersburg State I. Pavlov Medical University, at COVID-19 specialized department from May to November 2020. They were diagnosed with COVID-19 (U07.1), and the virus was identified via qualitative PCR (detection of SARS-CoV-2 RNA). Blood samples were taken in the acute phase of the disease at the time of admission (5–14 days from the first symptoms without any signs of recovery). Among patients with current COVID-19, the sex ratio comprised 42.9% men and 57.1% women. Age median made up 60 with standard deviation ± 17. All individuals in this cohort presented typical COVID-19 associated symptoms, i.e., fever, sense of fatigue, muscle and joint pains, cough and pneumonia confirmed by CT-scans. Out of all 64 patients with COVID-19, 69.1% were diagnosed with moderate course of the disease, and 30.1% with severe course, based on the criteria provided by guidelines of Russian Ministry of Health on COVID-19 diagnosis and treatment. By the time of their discharge, only 3.75% of all patients had completely recovered from the infection, 50% showed positive dynamic in their general condition as well as their CT-scan results, yet 38.8% were discharged without any significant positive changes in their lung tissues. Smaller percentage of patients with acute COVID-19 (5%) passed away due to infection complications.

Convalescents’ sex ratio comprised 47.3% men and 52.63% women, with age median being 32 ± 10. All of them had recently recovered from COVID-19, medians for the numbers of days since their first positive PCR and their official discharge being 47 ± 20 and 35 ± 17, respectively. Out of this cohort, 47.4% presented mild COVID-19 course of the disease, 40.4% were diagnosed with moderate course of the disease and 12.3% had recovered from severe COVID-19.

Healthy donors cohort consisted of 44 healthy individuals (20 male and 24 female), and their blood samples were collected prior the COVID-19 pandemic. Because of the high median age of the patients with COVID-19 it was problematic to form the control group of healthy donors with comparable age that lacked comorbidities. That is why subjects from the healthy donors group had significantly lower median age 40 (34; 49) years comparing to the patients. That was the limitation of the present study. 

### 2.2. Sample Collection

All experiments were performed within few hours (≤6 h) after blood collection. Peripheral blood samples were collected into vacuum test tubes added with K3-EDTA anti-coagulant (followed by processing for analyzing the relative and absolute counts of major T and B cell subsets with multicolor flow cytometry).

### 2.3. Flow Cytometry B Cell Immunophenotyping

B cell whole peripheral blood samples (200 μL) were stained with the following anti-human monoclonal antibodies IgD-Alexa Fluor 488 (cat. 348216, BioLegend, Inc., San Diego, CA, USA), CD38-PE (cat. A07779, Beckman Coulter, Brea, CA, USA), CD5-ECD (cat. A33096, Beckman Coulter, USA), CD27-PC7 (cat. A54823, Beckman Coulter, Brea, CA, USA), CD19-APC/Cy7 (cat. 302218, BioLegend, Inc., USA), and CD45-Krome Orange (cat. A96416, Beckman Coulter, USA), all antibodies were utilized at the dilutions that were recommended by the manufacturers. After incubation at room temperature in the dark for 10 min, erythrocytes were lysed for 15 min by adding 2 mL of VersaLyse Lysing Solution (Beckman Coulter, Inc., USA) supplied with 50 μL IOTest 3 Fixative Solution (Beckman Coulter, Inc., USA). Next, cells were washed (7 min, 330 g) twice with a buffer (sterile phosphate-buffered saline (PBS) containing 2% of heat inactivated fetal bovine serum, Sigma-Aldrich, St. Louis, MO, USA) and were resuspended in 0.5 mL PBS containing 2% of neutral buffered formalin solution (Sigma-Aldrich, St. Louis, MO, USA). Sample acquisition was performed using a Navios flow cytometer (Beckman Coulter, Inc., USA), equipped with 405, 488 and 638 nm lasers. There were collected at least 5000 CD19+ B cells to be analyzed in each sample. 

### 2.4. T Cell Immunophenotype by Flow Cytometry

Eight-color flow cytometry was used to analyze the surface phenotype (CD3, CD4, CD45RA and CD62L) and chemokine receptors (CXCR5, CCR6, CXCR3 and CCR4) on peripheral blood lymphocytes. The antibodies used were as follows: for surface phenotyping was performed with CD45RA-FITC (cat. IM0584U, Beckman Coulter, USA), CD62L-PE (cat. IM2214U, Beckman Coulter, USA), CD3-APC-AF750 (cat. A94680, Beckman Coulter, USA) and CD4-PacB (cat. B49197, Beckman Coulter, USA), whereas chemokine receptor profile was assessed by using CXCR5-PerCP/Cy5.5 (CD185, cat. 356910, BioLegend, Inc., USA), CCR6-PE/Cy7 (CD196, cat 353418, BioLegend, Inc., USA), CXCR3-APC (CD183, cat. 353708, BioLegend, Inc., USA), and CCR4-BV510 CD194, cat. 359416, BioLegend, Inc., USA). Staining protocols were performed in accordance with the manufacturer’s recommendations. In brief, 200 μL of whole peripheral blood sample were stained with antibody cocktail noted above (all antibodies were utilized at the dilutions that were recommended by the manufacturers) in the dark at room temperature for 15 min, followed by erythrocyte lysis with 2 mL of VersaLyse Lysing Solution (Beckman Coulter, Inc., USA) added with 50 μL IOTest 3 Fixative Solution (Beckman Coulter, Inc., USA; incubation time—15 min in the dark at room temperature). Next, all samples were washed once with PBS and centrifuged for 7 min at 330× *g*, resuspended in 500 μL of PBS added with 2% of neutral formalin (cat. HT5011-1CS, Sigma-Aldrich Co., USA) and, finally, analyzed by flow cytometry with a Navios flow cytometer (Beckman Coulter, USA). At least 40,000 CD3+CD4+ Th cells were collected for each sample. The data collected were analyzed with the Kaluza software (Beckman Coulter, Inc., USA).

### 2.5. Statistical Analysis

The flow cytometry data were analyzed with Kaluza 2.0 software (Beckman Coulter, Inc., USA). All of the statistical analysis of data was carried out with STATISTICA Version 8.0 (StatSoft Inc., Tulsa, OK, USA) and GraphPad Prism Version 5.0 (USA). The obtained data were tested for normality of distribution using the Shapiro-Wilk test (НС group contained less then 50 patients). The statistical comparisons of data between patients with acute COVID-19, convalescent patients and HC were performed using the Mann–Whitney U test. The differences between the groups were considered significant when *p* values were <0.05.

## 3. Results

### 3.1. Alterations in Peripheral Blood B Cell Subset Composition of COVID-19 Patients

Analyzing the total CD19+ B cell fraction of peripheral blood lymphocytes, we revealed no significant difference between the three cohorts. The relative number of circulating B cells in acute COVID-19 group was 13.29 ± 0.73%, lymphocyte fraction blood samples from convalescent patients contained 11.45 ± 0.47% B cells, whereas in healthy control group the frequency of CD19-expressing lymphocytes was 12.64 ± 0.53%.

Next, using multicolor flow cytometry, we assessed the percentage of circulating B cell subsets using a so-called “Bm1-Bm5” classification—the one of the crucial classification schemes based on the relative expression of surface IgD and CD38 markers [11]. Thus, IgD and CD38 staining was used to identify “naïve” IgD+CD38− Bm1 cell, “activated naïve” Bm2 cells (IgD+CD38+), pre-germinal-center Bm2′ cells (IgD+CD38++), a common subset consisting of centroblasts and centrocytes (so-called “Bm3+Bm4” cells, IgD-CD38++), as well as early memory and resting memory cells (eBm5 and Bm5 cells with the following phenotypes—IgD-CD38+ and IgD-CD38−, respectively). The data obtained are summarized in Table 1. We observed significantly decreased level of memory B cell subsets and increased proportion of centroblasts and centrocytes in patients with acute COVID-19 compared to healthy controls and COVID-19 convalescent patients. The relative numbers of virgin naïve, Bm2′ and “Bm3+Bm4” cells in COVID-19 convalescent patients were elevated compared with HC. 

We classified B cell subsets by using CD27 and CD38 co-expression to identify the following B cell subsets [12]: transitional B cells (CD27−CD38++), mature naive B cells (CD27−CD38+), mature activated B cells (CD27+CD38+), plasmablasts (CD27++CD38++), resting memory B cells (CD27+CD38−), and double-negative B cells (CD27−CD38−). The data obtained are summarized in Table 2. It was shown that mature active and memory B cell frequencies were decreased in patients with acute COVID-19 that significantly differed from the pattern found in healthy controls and COVID-19 convalescent patients, whereas the level of circulating plasmablasts/plasma cells was increased. Furthermore, COVID-19 convalescent patients had higher percentage of activated B cell subsets—circulating plasmablasts and transitional B cells—compared to healthy donors. 

Taken together, these results indicate that peripheral blood B cells from patients with acute COVID-19 demonstrated the aberrant distribution of ‘naïve’, memory and antibody-secreting plasmablasts/plasma cells.

### 3.2. Tfh Subset Imbalance in COVID-19 Patients

**Figure 1:**
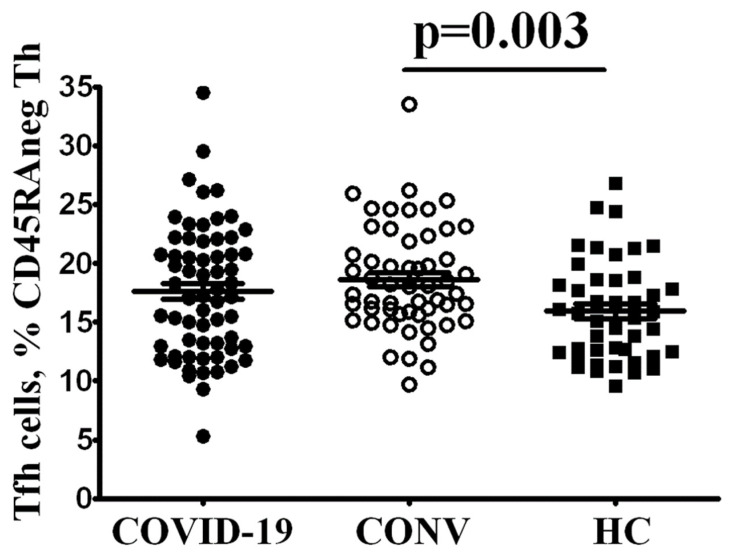
Evaluated level of circulating memory Tfh cells in COVID-19 convalescent patients.Scatter plots showing the percentages of CXCR5-expressing Th cell among total CD3+CD4+CD45RA− memory Th cells in the peripheral blood samples from patients with acute COVID-19 (COVID-19, black circles, *n* = 64), COVID-19 convalescent patients (CONV, white circles, *n* = 55) and healthy control (HC, black square, *n* = 44). Each dot represents individual subjects. and horizontal bars depict the group mean and standard error of the mean (Mean ± SEM). Statistical analysis was performed with the Mann-Whitney U test.

Because the rate of several B cell subsets was markedly altered, we decided to analyze the frequency and phenotype of follicular Th cells that control all stages of B cell differentiation and activation occurring in peripheral lymphoid tissues. It is known that circulating Tfh cells display a “central memory” or “memory” phenotype, predominantly reside in the secondary lymphoid organs and can recirculate in peripheral blood [13]. Primarily, we estimated the CXCR5+ Tfh frequencies within CD3+CD4+CD45RA− memory cell subset and found that COVID-19 convalescent patients had elevated Tfh cell count compared with HC (Figure 1), reflecting the expansion of cell type after acute phase of SARS-CoV-2 infection. A trend toward higher proportions of circulating Tfh was noted in patients with acute COVID compared to controls, but did not reach statistical significance. 

Currently, no standard marker panel for Tfh subsets analysis is available, and different researchers apply several approaches for Tfh subsets identification. However, it is well-known that circulating Tfh cells express chemokine receptors corresponding to the polarized non-Tfh cell subsets such as Th1, Th2 and Th17 cells [14]. Thus, the chemokine receptor CXCR3 and CCR6 co-expression allowed to identify the four functionally distinct Tfh subsets: CXCR3+CCR6− Tfh1, CXCR3−CCR6− Tfh2, CXCR3−CCR6+ Tfh17 and CXCR3+CCR6+ DP Tfh cells.

**Figure 2:**
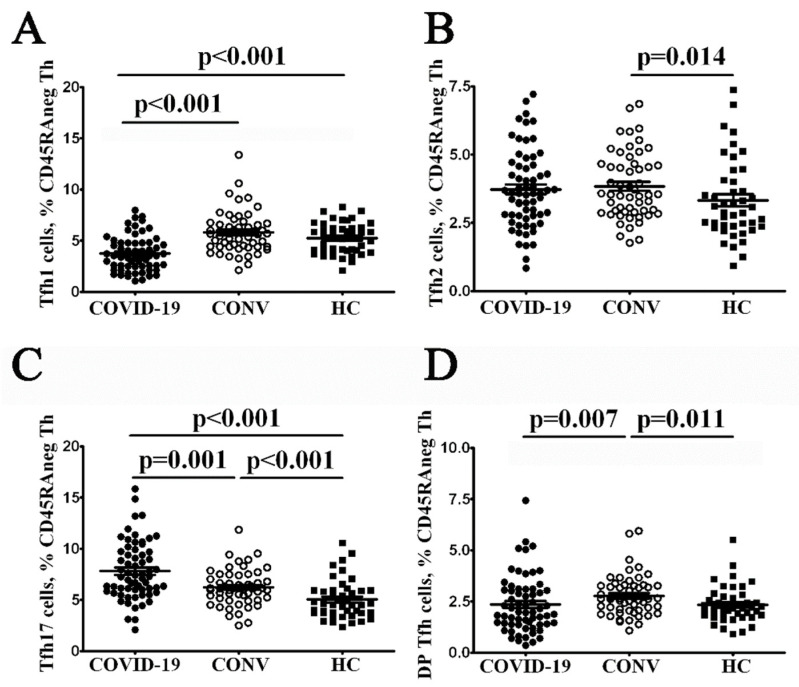
Imbalanced Tfh cell subsets in patients with acute COVID-19 and COVID-19 convalescent patients. From left to right: scatter plots showing the percentages of CXCR3+CCR6− Tfh1-like (**A**), CXCR3−CCR6− Tfh2-like (**B**), CXCR3−CCR6+ Tfh17-like (**C**) and unclassified double-positive CXCR3+CCR6+ T cell subsets among total CD3+CD4+CD45RA− T cell population (**D**), respectively, in the peripheral blood samples from patients with acute COVID-19 (COVID-19, black circles, *n* = 64), COVID-19 convalescent patients (CONV, white circles, *n* = 55) and healthy control (HC, black square, *n* = 44). Each dot represents individual subjects. and horizontal bars depict the group mean and standard error of the mean (Mean ± SEM). Statistical analysis was performed with the Mann-Whitney U test.

Our overall analysis of such Tfh cell subsets patients with acute COVID-19 indicated that CXCR3+CCR6− Tfh1-like cells were significantly decreased compared with healthy control and COVID-19 convalescent patients 3.74 ± 0.21% vs. 5.79 ± 0.27% (*p* < 0.001) and 5.09 ± 0.23% (*p* < 0.001), respectively (Figure 2). Furthermore, patients with acute COVID-19 had elevated levels of CXCR3−CCR6+ Tfh17-like cells that significantly differed from those found in healthy controls and COVID-19 convalescent patients 7.80 ± 0.35% vs. 5.01 ± 0.27% (*p* < 0.001) and 6.21 ± 0.23% (*p* = 0.001), respectively. Finally, we found several circulating Tfh subsets alterations in COVID-19 convalescent patients. Though, the relative numbers of Tfh1-like cells were invariable, we noticed the increased levels of all other Tfh subsets—Tfh2-, Tfh17- and DP Tfh-like cells—while comparing their amount in HC. 

Thus, such observations suggest that both groups of patients had aberrant distribution of circulating memory Tfh cell subsets and imbalanced Tfh cell subsets that might be associated with abnormal distribution of peripheral blood B cell subsets noted above. 

## 4. Discussion

The data obtained in this study regarding a relative number of total B cell pool in peripheral blood of COVID-19 patients points at lack of prominent differences compared with control groups that agrees with the data obtained by other researchers [15,16]. At the same time, a sufficient number of publications evidencing about decreased level of relative and absolute number of circulating B cell subsets has been reported [4,17,18,19]. Moreover, some studies suggest that increased number of CD19+ B cells was found in patients with severe vs. mild-to-moderate COVID19 infection [15], whereas others evidence about significantly reduced proportion of B cells mirroring escalating severity of COVID-19 [18]. 

Along with that, we noted that patients with acute COVID-19 had markedly altered B cell subset composition that was related to lowered proportion of circulating memory B cells and increased percentage both of activated and effector cells. Available publications suggest that patients with COVID-19 had significantly lowered percentage of circulating memory B cells regardless of disease severity [15]. Patients with severe and critically ill course of COVID-19 had lowered percentage of memory IgD+CD27+ B cells, whereas solely severe patients had profoundly decreased in memory IgD−CD27+ B cell subset compared to healthy volunteers. Our observation agrees with other studies [19,20,21,22], repeatedly suggesting about altered maturation and generation of memory B cells occurring in the secondary lymphoid tissues. Moreover, based on analyzing the key B cell subsets we found that critically ill patients were characterized by elevated percentage of CD27+CD38hi, CD21−CD11c−DN3 and IgD−CD27+ memory B cells, whereas patients with severe COVID-19 had elevated percentage of CD21−CD11c+ DN2, atypical CD27-negative memory B cells as well as CD11c+IgD+CD38−/+ activated naïve B cells [15]. Moreover, we also noted a decreased proportion of naïve IgD+CD38− B cells in the circulation that may point at altered B cell differentiation in the red bone marrow. Similar data were also obtained by Kaneko et al. showing that patients with COVID-19 had lowered peripheral blood relative and absolute number of naïve IgD+CD27− cells, transitional IgD+CD27−CD10+CD45RB− and follicular CXCR5+ (IgD+CD27−CD10−CD73+) B cells compared with control subjects or convalescent patients [20]. 

Regarding circulating effector B cells, it was found that COVID-19 patients had upregulated level of Ki67 marker as well as surface activation marker CD95 in circulating CD27+CD38+CD138+ B cells that might suggest about recent emigration from germinal center of the secondary lymphoid tissues [22]. Moreover, comparing groups of patients with varying intensity of COVID-19 it was shown that severe disease was featured with more pronounced decline in circulating B cells albeit it was not confirmed in other reports [23]. At the same time, a significantly elevated proportion of circulating CD19+CD20−CD38highCD27high plasma blasts was a common trait for all COVID-19 patients regardless of disease severity. However, a substantial percentage of circulating RBD-specific plasma blasts was found even in the acute phase of COVID-19 [24]. Hence, high percentage of circulating plasma cell level points not only at high intensity of antigen-specific B cell differentiation, but also potential alterations in control over such events applied by T follicular helper cells. Moreover, even convalescent patients we noted to have elevated level of circulating precursor plasma cells in peripheral blood that full recovery of immune system does not occur even some time later after SARS-CoV-2 infection. 

Altered B cell subset composition may be related to changes in Tfh cell functional activity inferred to aid in developing B cell-dependent humoral immune response [9,10]. We demonstrated that elevated proportion of circulating Tfh cells was noted solely in convalescent patients, whereas its level in control group did not differ from that found in patients with acute COVID-19. In particular, percentage of circulating Tfh cell in COVID-19 was lowered regardless of disease severity [25], but within CD45RA−CD62L+ central memory Th cells the lower frequencies of Tfh-like cells were detected in patients with severe infection compared to those in patients with moderate infection [19]. Although some studies noted lack of differences between healthy volunteers vs. COVID-19 patients [26] or elevated percentage of circulating Tfh cells [27]. Interestingly, peripheral blood of patients recovered after COVID-19 had no changes in peripheral blood Tfh cell percentage compared to control group, whereas proportion of CXCR5+PD-1highCD4+ Tfh and CCR7loPD-1+ effector memory T follicular cells (Tfh-cm) was increased in parallel with lowered count of CCR7hiPD-1− central memory T follicular cells able to migrate to the secondary lymphoid tissues [28]. 

We also found altered CD45RA−CXCR5+ T cell subset composition in patients with acute COVID-19 as well as convalescent subjects. Earlier it was shown that peripheral blood Tfh cell subsets exerted helper properties and might be divided into several populations based upon expression of surface chemokine receptors CCR6 and CXCR3 [14]. In particular, Tfh2 and Tfh17 (CXCR3−CCR6− and CXCR3−CCR6+, respectively) are able to stimulate naïve B cells to switch produced antibi classes and immunoglobulin secretion (from IgM to IgG and IgE or IgG and IgA, respectively), whereas memory CXCR3+ Tfh1 cells exert no such stimulatory effect, but rather may induce apoptosis in activated naïve B cells. In particular, we found that patients with acute COVID-19 had lowered percentage of regulatory Tfh1 cells along with elevated pro-inflammatory Tfh17 cell subset. The data reported by others suggest that peripheral blood of patients recovered after COVID-19 had elevated proportion of CXCR3+CCR6− Tfh1 and CXCR3−CCR6− Tfh2 cells compared with control group, whereas percentage of CXCR3−CCR6+ Tfh17 cells was significantly decreased [28]. Likewise, previously we shown the increased levels of two CXCR3 ligands—CXCL10/IP-10 and CXCL9/MIG—in blood plasma from COVID-19 patients during acute phase of the disease (as compared to control group) [9], and high levels of plasma chemokines could affect the biological properties of these CXCR3-expressing cells. 

Finally, we noted that patients successfully recovered after SARS-CoV-2 infection had elevated all effector Tfh cell subsets primarily peripheral blood Tfh2 and Tfh17 cells suggesting about profound alterations in functioning of antigen-specific immunity after COVID-19. At the same time, convalescent subjects had high antibody-neutralizing serum potential correlating with high proportion of cTfh1 and cTfh2 cells [29]. Moreover, subjects recovered after COVID-19 contained circulating virus-specific CD45RA−CXCR5+ Tfh cells able to recognize viral S-protein, whereas proportion of RBD-specific Tfh cells was extremely low [29]. Moreover, the vast majority of SARS-CoV-2-specific Tfh cells also consisted of CCR6+CXCR3− Tfh17 cells, some of which displayed Tfh1 (CCR6−CXCR3+) cell phenotype. The association of phenotypic changes in circulation Tfh subsets with different clinical forms of COVID-19 [19] and, especially, in patients successfully recovered after SARS-CoV-2 infection suggests the potential contribution of T follicular helper cells as features or risk factors for post-COVID-19 symptoms and diseases [30,31,32].

It was showed that the level of circulating plasmablast precursors was peaked consistently at day 6 or 7 after acute virus infection and then dropped to baseline levels within 2–3 weeks post symptom onset [33]. For instance, plasmablast (co-expressing CD27 and CD38 CD19+ cells) frequencies were 10–50% of B cells in patients infected with Ebola virus, compared with less than 1% in healthy individuals [34]. Next, dengue-infected patients the frequency of antibody secreting cells was significantly increased and associated with the lower frequency of naïve B cells in peripheral blood [35]. Moreover, frequency of plasmablasts significantly and positively correlated with the frequency of the total Tfh cells during the acute phase of dengue virus infection [36]. Furthermore, the frequency of Tfh cells was significantly higher during the convalescent phase compared to the frequencies in acute phase and in healthy individuals. Interestingly, vaccine-induced plasmablast (circulating CD27+CD38++CD20− B cells) expansion that followed influenza virus vaccination was detected in peripheral blood 7 days after vaccination [37]. Similarly, it was shown that PD-1+ICOS+ circulating Tfh cell subsets at day 7 correlated with plasma specific IgG functional affinity at their peak levels at days 14 and 21 [38]. 

Conclusions. Taken together these data indicates that the presence of high frequencies of circulating plasma cell precursors and altered Tfh cell subsets could be closely linked with acute viral infections and might constitute residual effects by which COVID-19 can impact the homeostasis of B cell and Tfh cell interactions in the long-term. Furthermore, the better understandings of these long-term alterations in adaptive humoral immunity (which required participation from different types of B cells and CD4+ T cells) in COVID-19 convalescent patients could help us to develop long-term host protection against SARS-CoV-2, effective secondary response against future SARS-CoV-2 infections and predict the generation of a highly effective humoral and cellular immunity in response to vaccination [39]. Many of these questions linked with SARS-CoV-2-specific immunity require further studies.

## Figures and Tables

**Table 1:** The percentage of B-cell subsets assessed by using “Bm1-Bm5” classification in patients with acute COVID-19 (COVID-19, *n* = 64), COVID-19 convalescent patients (CONV, *n* = 55) and healthy control (HC, *n* = 44). The quantitative data (% with CD19+ subset) are presented as mean and standard error of the mean (Mean ± SEM).

B Cell Subset	Phenotype	COVID-19	CONV	HC	Significant Differences
Bm1	IgD+CD38−	12.28 ± 1.23	15.83 ± 0.62	12.39 ± 0.73	p_1_ < 0.001 p_2_ = 0.129 p_3_ = 0.004
Bm2	IgD+CD38+	57.79 ± 1.91	52.84 ± 1.06	56.83 ± 1.32	p_1_ = 0.001 p_2_ = 0.165 p_3_ = 0.040
Bm2′	IgD+CD38++	9.98 ± 0.79	11.57 ± 0.77	8.97 ± 0.57	p_1_ = 0.109 p_2_ = 0.919 p_3_ = 0.044
Bm3+Bm4	IgD−CD38+++	6.00 ± 0.68	2.16 ± 0.20	1.28 ± 0.16	p_1_ < 0.001 p_2_ < 0.001 p_3_ < 0.001
eBm5	IgD−CD38+	7.47 ± 0.72	9.26 ± 0.44	10.93 ± 0.75	p_1_ < 0.001 p_2_ < 0.001 p_3_ = 0.125
Bm5	IgD−CD38−	6.49 ± 0.63	8.34 ± 0.48	9.60 ± 0.79	p_1_ < 0.001 p_2_ < 0.001 p_3_ = 0.267

Note: p_1_—statistical differences between patients with acute COVID-19 and COVID-19 convalescent patient groups according to nonparametric Mann-Whitney U tests; p_2_—statistical differences between patients with acute COVID-19 and healthy control groups according to nonparametric Mann-Whitney U tests; p_3_—statistical differences between COVID-19 convalescent patients and healthy control groups according to nonparametric Mann-Whitney U tests.

**Table 2:** The percentage of B-cell subsets assessed by using CD27 vs. D38 classification in patients with acute COVID-19 (COVID-19, *n* = 64), COVID-19 convalescent patients (CONV, *n* = 55) and healthy control (HC, *n* = 44). The quantitative data (% with CD19+ subset) are presented as mean and standard error of the mean (Mean ± SEM).

B Cell Subset	Phenotype	COVID-19	CONV	HC	Significant Differences
Naive mature	CD27−CD38+	12.28 ± 1.23	15.83 ± 0.62	12.39 ± 0.73	p_1_ < 0.001 p_2_ = 0.129 p_3_ = 0.004
Mature active	CD27+CD38+	57.79 ± 1.91	52.84 ± 1.06	56.83 ± 1.32	p_1_ = 0.001 p_2_ = 0.165 p_3_ = 0.040
DN cells	CD27−CD38−	9.98 ± 0.79	11.57 ± 0.77	8.97 ± 0.57	p_1_ = 0.109 p_2_ = 0.919 p_3_ = 0.044
Memory	CD27+CD38−	6.00 ± 0.68	2.16 ± 0.20	1.28 ± 0.16	p_1_ < 0.001 p_2_ < 0.001 p_3_ < 0.001
Plasmablasts	CD27++CD38+	7.47 ± 0.72	9.26 ± 0.44	10.93 ± 0.75	p_1_ < 0.001 p_2_ < 0.001 p_3_ = 0.125
Transitional cells	CD27−CD38++	6.49 ± 0.63	8.34 ± 0.48	9.60 ± 0.79	p_1_ < 0.001 p_2_ < 0.001 p_3_ = 0.267

Note: p_1_—statistical differences between patients with acute COVID-19 and COVID-19 convalescent patient groups according to nonparametric Mann-Whitney U tests; p_2_—statistical differences between patients with acute COVID-19 and healthy control groups according to nonparametric Mann-Whitney U tests; p_3_—statistical differences between COVID-19 convalescent patients and healthy control groups according to nonparametric Mann-Whitney U tests.

## Data Availability

Not applicable.
